# Eating for one, exercising for two: a long journey from old knowledge to new perspectives

**DOI:** 10.1007/s10654-016-0204-0

**Published:** 2016-10-08

**Authors:** Ylva Trolle Lagerros

**Affiliations:** 1 Clinical Epidemiology Unit, Department of Medicine, Karolinska Institutet, T2, 171 76 Stockholm, Sweden; 2 Department of Endocrinology, Metabolism and Diabetes, Karolinska University Hospital, Huddinge, Stockholm, Sweden

Almost a hundred years ago, back in 1922, when undernutrition was a larger concern than obesity, the authors of the *Statistical Bulletin of the Metropolitan Life Insurance Company* concluded, as a final remark, that: “The important lesson taught… is that there is an optimum build with reference to mortality. The average build is not the best build. Those who weigh between 10 and 20 % below average show the optimum condition of longevity at most of the ages after early adult life” [1].

These initial observations in life insurance data, showing that individuals with a normal body weight lived longer than overweight clients, have during the last decade been followed by numerous epidemiological studies on body mass index (BMI) and mortality. The message, that obesity is hazardous to health, initially fell on deaf ears. Today, however, when overweight is more common than underweight, even in low- and middle-income countries, the same message has impacted public perceptions to such a degree that we are bombarded with headlines including expressions like “obesity epidemic”. Meanwhile, research and epidemiologists have moved on. We are now not only studying obesity and adult mortality, but obesity in one generation and mortality of the next.

In this issue of the *European Journal of Epidemiology*, the results of two complementary investigations shed light on a potential window of opportunity for lifestyle intervention—pregnancy. Morales-Suárez-Varela et al. [2] primarily examined the impact of parental smoking on fetal death in the large population-based Danish National Birth Cohort. The hazards of smoking during pregnancy have been proven before, but Morales-Suárez-Varela et al. present novel results from interaction analyses with obesity and physical activity. Aune et al. [3] conducted a systematic review of epidemiological studies on physical activity and the risk of gestational diabetes, including a dose–response meta-analysis which also is presented in this issue of the journal.

Pre-pregnancy overweight and obesity have repeatedly been shown to increase the risk of complications during pregnancy. A meta-analysis including more than 10,000 fetal deaths, revealed that for each 5-unit increase in maternal BMI, the summary adjusted relative risk for fetal death increased with 21 % [4]. Further, a population-based study using the Swedish Medical Birth Register has reported that women gaining weight between their first and second pregnancy had a higher risk of stillbirth and infant mortality during their second pregnancy compared to their first [5]. Weight loss between the pregnancies was, on the contrary, shown to reduce the risk of neonatal mortality in the offspring of overweight women.

Physical activity is a modifiable factor that can prevent premature mortality in the general population, but that has not been convincingly documented to be associated with fetal death. This may in part be due to the fact that registers set up for health care commonly do not include behavioral factors such as physical activity, and thus, register-based studies lack this information. Morales-Suárez-Varela et al. solved this issue by putting an astonishing amount of work into conducting telephone interviews with the 87,930 pregnant women in the cohort.

Overall, in the study by Morales-Suárez-Varela et al., smoking during pregnancy was associated with a higher hazard ratio (HR) for fetal death if both parents smoked compared to non-smoking parents; HR 1.22 (95 % CI 1.02–1.46). There was also evidence of an additive interaction between smoking and high BMI, suggesting that smoking may increase the negative effect of a high BMI on fetal death. No interaction was observed between smoking and exercise.

Despite the impressive effort to assess both exposure and confounders by conducting telephone interviews in this large study, the authors may regret that physical activity was rudimentary assessed with the question: “Do you get any kind of exercise during pregnancy?”. Implicit in epidemiological research is the ability to estimate the strength of an association between exposure and outcome with as little error as possible. Accurate measurement is therefore central. Physical activity is complex to measure as it is a behavior that can take many forms. Exercise is only one type of physical activity behavior and activities in different domains or of different intensities may have different effects.

Aune et al. [3], who conducted the meta-analysis of physical activity and the risk of gestational diabetes, published in this issue of the journal, required included studies to have a quantitative measure of activity level with three or more categories to be eligible for their dose–response analysis. Twenty-six publications were included and then divided into subgroups of total-, leisure time-, occupational-, and household physical activity, undertaken before and during pregnancy. The dose–response analysis of leisure time activity reviled nonlinear inverse associations between physical activity before pregnancy, as well as physical activity in early pregnancy, and gestational diabetes. There was a steeper inverse association in the lower activity levels, indicating that the beneficial effect was evident even in those with low levels of activity compared with sedentary women.

Gestational diabetes, i.e. glucose intolerance discovered for the first time during pregnancy, is one of the most common pregnancy complications. It is associated with a number of adverse health outcomes for both the mother and the offspring. Results from previous studies have been conflicting, some suggesting no association and some suggesting that a higher level of physical activity is associated with a lower risk of gestational diabetes. The strengths of associations have spanned a wide range and decreased risks between 10 and 90 % have been shown. Three previous meta-analyses on the topic [6–8] came to different conclusions. They reported no vs. inverse associations between physical activity before or in early pregnancy and the risk of gestational diabetes, and none of them included a dose–response analysis. The new meta-analysis by Aune et al. adds to the existing literature as an analysis of a potential dose–response relationship between physical activity and gestational diabetes was also performed.

Pregnancy is no longer a contraindication for physical activity, but is rather recognized as a window of opportunity for behavior modification. It is safe to continue or start most types of exercise, according to the American College of Obstetricians and Gynecologists (ACOG) which encourages pregnant women with uncomplicated pregnancies to undertake regular physical activity during pregnancy [9]. Unfortunately, there is a lack of robust antenatal trials focusing on lifestyle, including physical activity, and dietary counseling. Most studies are small and have failed to prove a positive effect on clinically relevant outcomes such as maternal gestational diabetes. The largest randomized study to date included 2152 women with a BMI above 25, comparing standard care to lifestyle advise delivered by dieticians a number of times throughout pregnancy. The intervention was successful in reducing the risk of high infant birth weight, but failed to show an effect on gestational diabetes or weight gain during pregnancy [10]. One can speculate that a more intense intervention would have proven effective, but compliance and costs would likely have compromised feasibility. Improvements in adverse pregnancy outcomes, more common among overweight and obese women, seem challenging to achieve with lifestyle changes. Thus, the task of designing effective intervention studies is therefore a challenging one. Are we too late, trying to intervene in the pregnant woman? Is the window of opportunity really there, or should we start much earlier?

Women’s and children’s health have undergone immense improvements during the last generation. Nevertheless, the century old advice, to weigh below average, still holds true. The worldwide prevalence of obesity has doubled since 1980 and is not only a high-income country concern, according to WHO [11]. Globally, 40 % of the adult women are overweight [11], many of which will eventually become pregnant. The articles by Morales-Suárez-Varela et al. and Aune et al., highlight the importance of a healthy lifestyle for a healthy pregnancy. Our next challenge is how to move on from here. When and how to intervene to ensure healthy pregnancies and decrease risks for both the pregnant woman and her child should be obvious priorities in future research.
